# Evidence for a Minimal Eukaryotic Phosphoproteome?

**DOI:** 10.1371/journal.pone.0000777

**Published:** 2007-08-22

**Authors:** Sander H. Diks, Kaushal Parikh, Marijke van der Sijde, Jos Joore, Tita Ritsema, Maikel P. Peppelenbosch

**Affiliations:** 1 Kinome Profiling Unit, Department of Cell Biology, University Medical Center Groningen, University of Groningen, Groningen, Groningen, The Netherlands; 2 Pepscan Presto, Lelystad, Flevoland, The Netherlands; 3 Phytopathology, Institute of Environmental Biology, Utrecht University, Utrecht, Utrecht, The Netherlands; The University of Birmingham, United Kingdom

## Abstract

**Background:**

Reversible phosphorylation catalysed by kinases is probably the most important regulatory mechanism in eukaryotes.

**Methodology/Principal Findings:**

We studied the *in vitro* phosphorylation of peptide arrays exhibiting the majority of PhosphoBase-deposited protein sequences, by factors in cell lysates from representatives of various branches of the eukaryotic species. We derived a set of substrates from the PhosphoBase whose phosphorylation by cellular extracts is common to the divergent members of different kingdoms and thus may be considered a minimal eukaryotic phosphoproteome. The protein kinases (or kinome) responsible for phosphorylation of these substrates are involved in a variety of processes such as transcription, translation, and cytoskeletal reorganisation.

**Conclusions/Significance:**

These results indicate that the divergence in eukaryotic kinases is not reflected at the level of substrate phosphorylation, revealing the presence of a limited common substrate space for kinases in eukaryotes and suggests the presence of a set of kinase substrates and regulatory mechanisms in an ancestral eukaryote that has since remained constant in eukaryotic life.

## Introduction

Kinases are enzymes that transfer a phosphate to an acceptor, which can be carbohydrates, lipids or proteins. The superfamily of eukaryotic protein kinases responsible for phosphorylation of specific tyrosine, serine, and threonine residues is generally recognised as the major regulator of virtually all metabolic activities in eukaryotic cells including proliferation, gene expression, motility, vesicular transport, and programmed cell death [1]. Dysregulation of protein phosphorylation plays a major role in many diseases such as cancer and neurodegenerative disorders, and characterisation of the human kinome space revealed that 244 of 518 putative protein kinase genes are currently mapped to disease loci or cancer amplicons [2], [3]. Accordingly, drugs targeting protein kinases are promising avenues for the therapeutic treatment of a plethora of different diseases [4]. In addition, elucidating kinase cascades has proved pivotal for understanding and manipulating cellular behaviour in a variety of divergent eukaryotes.

Most members of the protein kinase superfamily of enzymes can be recognized from their primary sequences by the presence of a catalytic eukaryotic protein kinase (ePK) domain of approximately 250 amino acids, whereas a small number of protein kinases do not share this catalytic domain and are often collectively called atypical kinases [5], [6]. A comparison of kinase domains both within and between species displays substantial diversity, which is further increased by the non-catalytic functional domains of kinases that are involved in regulation, interactions with other protein partners, or subcellular localisation. This diversity in catalytic and non-catalytic domains explains the functional diversification of kinases within the eukaryotic kingdom. Eukaryotic protein kinases are now generally classified into several major groups [7], [8]: the cyclic nucleotide- and Ca2+-/phoshospholipid-dependent kinases (AGC); a group consisting of the cyclin-dependent and cyclin-dependent-like kinases, mitogen-activated kinases, and glycogen synthase kinases (CMGC); the tyrosine kinases (TK); the tyrosine kinase-like group (which are in fact serine/threonine protein kinases) (TKL); the calmodulin-dependent kinases (CAMK); the casein kinase 1 group (CK); and the STE group (first identified in analyses of sterile yeast mutants) that includes the enzymes acting upstream of the mitogen-activated kinases (STE), summarised in table 1 which is an extension on the table published by Manning et al. 2002. Plants were considered not have a TK group but instead have a large receptor-like kinase group (RLK). However, recently Miranda–Saavedra *et al.* have shown using a new library that this is not the case. This new library is outperforms BLASTP and general Pfam hidden Markov models in the classification of kinase domains. They show that plants do contain tyrosine kinases and that diverse classes of organisms have a large overlap in kinase families [8]. It should be noted, however, that many eukaryotes also have kinase sequences that are not easily assigned to one of these groups and are referred to as “other protein kinases.” Thus far, pan-eukaryotic classification of kinase substrate sequences has not been attempted and would give better insight in the evolution and variability of substrates and their kinases.

**Table 1:** Classification of the different kinases in major groups and the numbers of members detected in different organisms by genetic screens (general estimates).

Class	Description	Yeast	Dictyostelium	Worm	Fly	Sea Urchin	Plant	Human
**AGC**	PKA, PKC, PKG	17 (13%)	21 (7%)	30 (7%)	30 (13%)	29 (8%)	43 (4%)	63 (12%)
**CAMK**	Calcium/calmodulin Kinases	21 (16%)	21 (7%)	46 (10%)	32 (13%)	50 (14%)	89 (9%)	74 (14%)
**CK1**	Casein Kinase	4 (3%)	2 (1%)	85 (19%)	10 (4%)	6 (2%)	18 (2%)	12 (2%)
**CMGC**	CDK, MAPK, GSK3, CLK	21 (16%)	28 (9%)	49 (11%)	33 (14%)	35 (10%)	65 (7%)	61 (12%)
**Other**		38 (29%)	71 (24%)	67 (15%)	45 (19%)	92 (26%)	19 (2%)	83 (16%)
**STE**	Homologues of sterile	14 (11%)	44 (15%)	25 (6%)	18 (8%)	21 (6%)	67 (7%)	47 (9%)
**TK**	Tyrosine Kinase	0 (0%)	0 (0%)	90 (20%)	32 (13%)	53 (15%)	0 (0%)	90 (17%)
**TKL**	Tyrosine Kinase-like	0 (0%)	68 (23%)	15 (3%)	17 (7%)	35 (10%)	52 (5%)*	43 (8%)
**RGC**	Receptor guanylate Cyclase	0 (0%)	0 (0%)	27 (6%)	6 (3%)	8 (2%)	0 (0%)	5 (1%)
**RLK/Pelle**	Receptor Like Kinases	0 (0%)	0 (0%)	0 (0%)	0 (0%)	0 (0%)	620 (64%)	0 (0%)
**Atypical**	PDHK	2 (2%)	0 (0%)	1 (0%)	1 (0%)	2 (1%)	0 (0%)	5 (1%)
**Alpha**		0 (0%)	6 (2%)	4 (1%)	1 (0%)	3 (1%)	0 (0%)	6 (1%)
**RIO**		2 (2%)	2 (1%)	3 (1%)	3 (1%)	3 (1%)	0 (0%)	3 (1%)
**TIF1**		1 (1%)	1 (0%)	2 (0%)	1 (0%)	1 (0%)	0 (0%)	2 (0%)
**Other**		2 (2%)	20 (7%)	1 (0%)	2 (1%)	8 (2%)	0 (0%)	9 (2%)
**ABC1**		3 (2%)	4 (1%)	3 (1%)	3 (1%)	4 (1%)	0 (0%)	5 (1%)
**Brd**		0 (0%)	2 (1%)	1 (0%)	1 (0%)	1 (0%)	0 (0%)	4 (1%)
**PIKK**		5 (4%)	5 (2%)	5 (1%)	5 (2%)	4 (1%)	0 (0%)	6 (1%)
**Total**		30 (100%)	295 (100%)	54 (100%)	240 (100%)	355 (100%)	973 (100%)	518 (100%)

References to the different kinomes are mentioned in the text. * In plants, this group consists only of raf-like members in the *A. thaliana* genome.

Comparative analyses of genomes have already demonstrated substantial differences in the kinomes of different eukaryotes. These differences are partly reflected in the highly variable number of protein kinase genes present in the genomes of different eukaryotes (*e.g.*, the *A. thaliana* genome contains 973 apparent protein kinases [9], the *H. sapiens* genome contains 518 [2], *S. purpuratus* is predicted to have 353 protein kinases [10] *D. melanogaster* appears to have 240 [7], *S. cerevisiae* has 115 protein kinase genes [11], and *P. falciparum* exhibits only 65 putative protein kinases) [12], as well as in highly divergent kinase structures. For instance, plant and unicellular eukaryotic genomes do not contain any apparent kinases from the tyrosine kinase group, despite the detection of phosphorylated tyrosine residues in plants, suggesting that tyrosine phosphorylation in these organisms is possible or that it is mediated via other types of kinases [13]–[16]. Strikingly, of the 106 putative protein kinases identified in *S. pombe* on the basis of primary sequence, only 67 have orthologues in *S. cerevisiea* but 47 have an orthologue in *H. sapiens* [17], indicating a great deal of conservation in kinases between different organisms. This high degree of overlap might indicate the presence of conservatism in kinase substrates too. In the *P. falciparum* kinome, 30% of protein kinases belong to the FIKK family of protein kinases that is apicomplexa-specific and not found in other groups of eukaryotes [12]. As mentioned previously, plants contain a large group of serine/threonine kinases (receptor-like kinases) not found in other eukaryotes. These RLKs most likely share a common evolutionary origin with the receptor tyrosine kinases present in animals and are thus sometimes collectively referred to as receptor kinases and providing an explanation that tyrosine containing motifs on the PepChip can be phosphorylated by these lysates [9]. Interestingly, a recent *in silico* report on the kinome of the sea urchin has provided new evidence on the evolution of different kinase subfamilies as being an intermediate eukaryote between animals and plants [10]. Fungi such as yeast and *Neurospora* do not appear to have representatives of the receptor kinase group, whereas the slime mould *D. discoideum* does have receptor kinases, which fits with the role of receptor kinases in multicellular organisms [18]. Thus, the eukaryotic family of protein kinases displays substantial diversity at the genetic level between different eukaryotic families.

Whether a kinase is able to phosphorylate its substrate depends on multiple factors such as the physical localisation of both molecules, availability of the substrate to the kinase, but a very important factor, in case of a protein kinase, is the amino acid context surrounding the phospho acceptor. The amino acids surrounding the substrate amino acid confer specificity to which kinase can bind correctly to the substrate and confer a phosphate group to the acceptor. The fact that different kinases have different target substrates is being exploited for phosphoproteome profiling using peptide arrays. In this approach, kinase substrates described in the PhosphoBase phosphorylation site database [19] are spotted on a glass slide and incubated with cell lysates and 33P-labelled γ-ATP. Phosphorylation of target peptides in arrays has provided substrate phosphorylation profiles for LPS-stimulated monocytes and was instrumental for the discovery of Lck and Fyn kinases as early targets of glucocorticoids [20], [21]. Importantly, the extent to which the diversity of kinases at the genetic level is reflected in differences in substrate specificity has not been investigated on a large scale.

In the present study, we investigated substrate requirements of phosphoproteomes of several divergent eukaryotes by employing peptide arrays on resting, unstimulated cellular lysates. Our results show that the divergence of eukaryotic protein kinases observed at the level of primary sequence is not completely reflected at the level of substrate phosphorylation, revealing a large overlap in the phosphorylation profiles from lysates of different eukaryotic origins. Furthermore, the identified minimal eukaryotic phosphoproteome suggests the presence of a set of kinase substrates in an ancestral eukaryote that has since remained invariant in eukaryotic life. The phosphoproteome seems to be involved in the maintenance of cell homeostasis as judged from the source of the peptides involved and thus may be a requisite for eukaryotic life [22].

## Results and Discussion

### Phosphorylation of peptide arrays exhibiting mammalian-biased kinase substrates by divergent eukaryote sources

**Figure 1:**
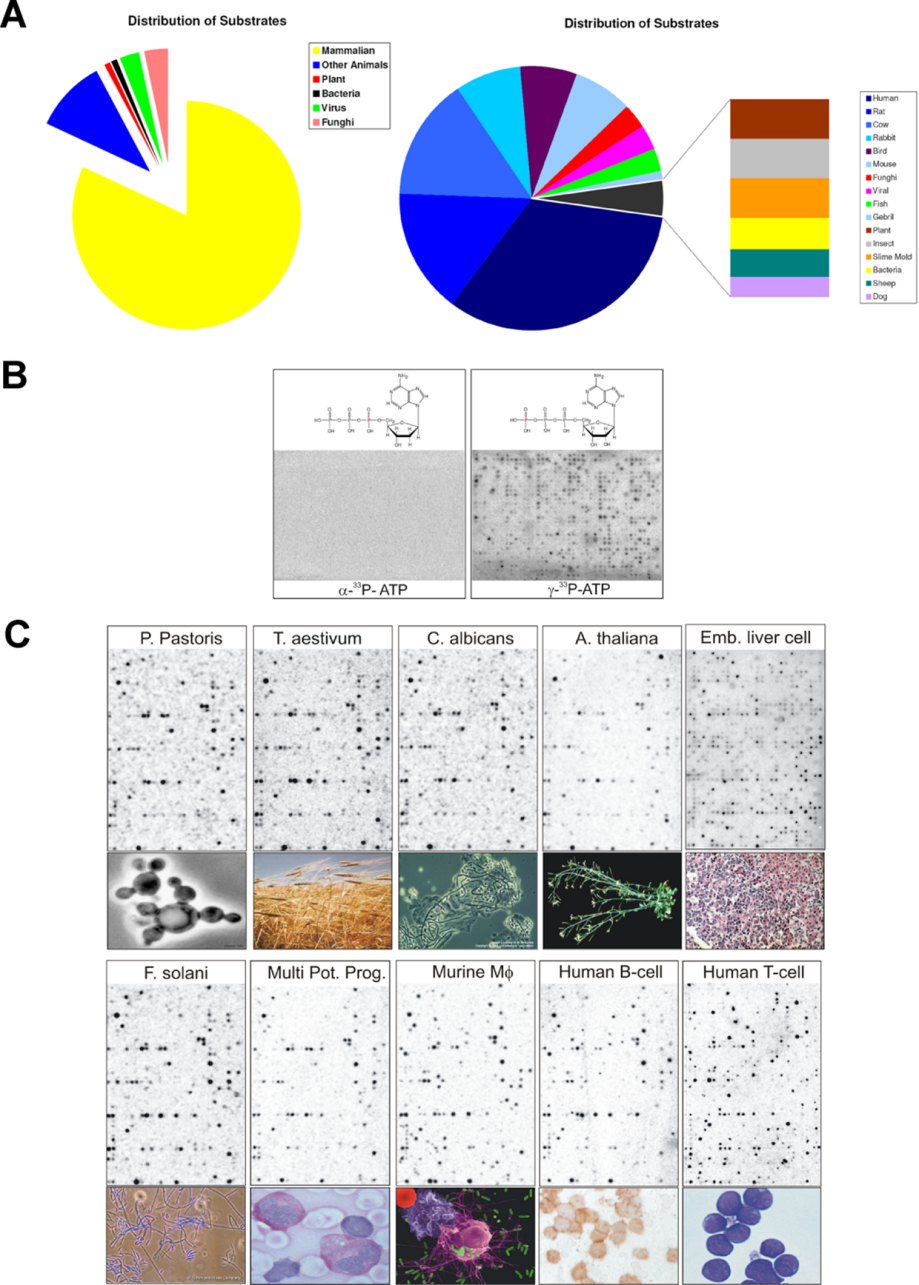
(A) Distribution of the primary origin of substrates spotted on the PepChip by regnum and species. (B) Incubation of a lysate on a PepChip with equal amounts of [^33^P-α]- and [^33^P-γ]-labelled ATP to show functional phosphorylation (C) Weighted average of at least three PepChip profiles of the different samples.

A peptide array (PepChip) was employed to determine the preference of cell lysates for kinase substrates. We used the PhosphoBase resource (version 2.0) (now called Phospho.Elm: http://phospho.elm.eu.org) as a source of diverse peptide substrates for kinases [19]. This database contains kinase substrate peptides from diverse organisms, including yeast and plant peptides, but is strongly biased towards mammalian peptide sequences (Figure 1A and Table S1). It must be noted that this set of substrates is just a small subset of known protein kinase substrates and the complete phosphoproteome which is considered to be a lot bigger. Arrays were constructed by covalently coupling chemically synthesized, soluble peptides to glass substrates as described previously [21]. Arrays contained 1152 different oligopeptides, covering the majority of substrate peptides available through PhosphoBase (version 2.0). On each carrier, the array was spotted twice to allow assessment of variability in substrate phosphorylation. The final physical dimensions of the array were 25×75 mm. Each peptide spot had a diameter of approximately 250 µm, and each spot was 620 µm from adjacent spots. When the arrays were incubated with [33P-γ] ATP and cell lysates from diverse eukaryotic sources, radioactivity was efficiently incorporated. In contrast, no radioactivity was incorporated when arrays were incubated with [33P-α] ATP and lysates, demonstrating that spot phosphorylation was mediated by specific attachment of the γ-phosphate of ATP to the oligopeptides in the array (Figure 1B). Both the technical replicates (same peptide on the same chip) and the biological replicates were generally of good quality (see supplementary data). Remarkably, the efficiencies by which cell lysates derived from divergent eukaryotic sources phosphorylated specific peptides in the array overlapped substantially, with mammalian lysates showing ^33^P incorporation in a large number of spots (Figure 1C). This overlap in phosphorylation of a strongly mammalian-biased set of kinase substrates indicates that a subgroup of kinases is present in divergent eukaryotes has similar peptide sequence requirements for catalysing phosphorylation reactions.

### Serine (S), threonine (T), and tyrosine (Y) phosphorylation is similar in divergent eukaryotes

**Figure 2:**
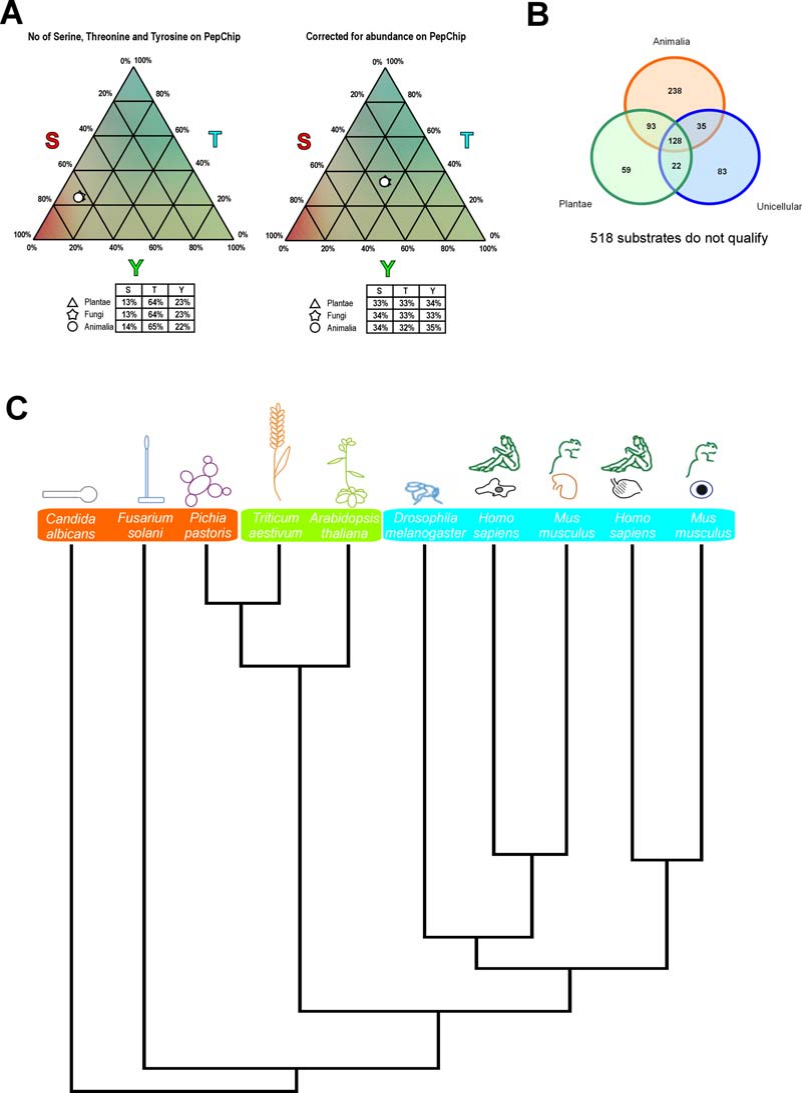
(A) Distribution of serine, threonine, and tyrosine substrates of the different phyla on the PepChip, before (left) and after (right) correction for the abundance of each phosphate acceptor on the PepChip. (B) Venn diagram of spots phosphorylated by the different regna. (C) Hierarchical clustering (according to Johnson [29]) of the phosphorylated motifs among the lysates tested.

Eukaryotic organisms from the plant and fungal kingdoms were not thought to express archetypical tyrosine kinases, as judged from the primary sequences of kinases present in their genomes. However, such organisms have been reported to be capable of phosphorylating tyrosine residues via dual-specificity kinases [11], [14]–[16], [23], [24]. Another explanation for tyrosine phosphorylation by these lysates is the fact that serine, threonine, and tyrosine are not the only phosphate acceptors in eukaryotes. Several lines of research have already shown that histidine and aspartate are also phosphorylated in eukaryotic cells (reviewed in [25]–[27]). Therefore, another explanation could be that histidine and/or aspartate kinases were a possible confounder in our minimal phosphoproteome set (Table 2). This is boosted by the observation that of the 353 monophospho-substrates, only 35% of the serine/threonine motifs contained a histidine (H) or aspartate (D) and 60% of the tyrosine motifs. The difference in the distribution of the H and D amino acids between S/T and Y containing motifs could imply that phosphorylation of histidine (H), aspartate (D) and tyrosine (Y) might have a common ancestry and a coupled evolutionary background which is not unlikely as remarkable similarities exists between these two classes of kinases (reviewed by Wolanin et al. and references therein)[28]. However, most the tyrosine substrates in our minimal phosphoproteome panel do not contain a histidine or aspartate and therefore common evolutionary backgrounds for histidine, aspartate and tyrosine seems less likely. Thus, the absence of obvious tyrosine kinases in the plant and fungal kingdoms does not result in the inability to phosphorylate tyrosine containing substrates in these organisms. Thus, we compared the relative capacities of animal-derived cell lysates to phosphorylate tyrosine-containing peptide substrates with lysates obtained from the other two eukaryotic kingdoms. To this end, we compared the contribution of serine, threonine, or tyrosine amino acid-containing substrates to the total phosphorylation of all peptide substrates, correcting for the relative abundance of the amino acid in the entire set of substrates. Peptides that can be phosphorylated at more than one residue would bias the results towards a particular amino acid. For example, a peptide that is phosphorylated at two adjacent serines could result in higher signal intensity than a peptide phosphorylated on one threonine. Thus, only those peptides with a single serine, threonine, or tyrosine phosphorylation site were considered (see Table S2). When array phosphorylation was studied in this manner, it appeared that the relative capacities of cell lysates to phosphorylate serine, threonine, or tyrosine substrates were remarkably similar, independent of the kingdom (Figure 2A and B).

**Table 2:** Distribution of the other phospho acceptors, histidine (H) and aspartate (D) in monophospho motifs (containing only one S, T or Y).

	All Substrates	Withou DH	With DH	%−DH	%+DH
**STY**	100% (353)	100% (219)	100% (134)	62%	38%
**ST**	87% (308)	92% (201)	80% (107)	65%	35%
**S**	69% (245)	72% (159)	64% (86)	65%	35%
**T**	18% (63)	19% (42)	16% (21)	67%	33%
**Y**	13% (45)	8% (18)	20% (27)	40%	60%

### Clustering of array phosphorylation patterns along phylogenetic lines

We wished to determine whether the patterns of array phosphorylation reflect phylogenetic relations among the various sources of the cell lysates. To this end, we calculated the Spearman correlation coefficient among the array results using all datasets separately (Table S3), combining datasets with similar origin (Table S4) or combining datasets to organisms (Table S5) and then clustered the results according to Johnson (Figure 2C) [29]. Histograms the distributions of positive spots of these three datasets analysis show a normal distribution which is shifted to the right (Histogram S1, S2 and S3). Cell lysates from plant and animal sources clustered *intra regna*, with plants showing less intraregnal variation than animals. This finding could arise from the fact that plant cell lysates were produced from entire organisms, whereas animal lysates were from specialised tissues. Strikingly, the variation in array phosphorylation was comparable between different human or different mouse lysates and between mammalian lysates and a Drosophila lysate. Substrate preferences for kinases do seem to have undergone some diversification after the separation of the animal and plant branches of the eukaryotes. For example, intraregnal variation in phosphorylation between monocotyledons and dicotyledons is smaller than the variation between *M. musculus* B-cells and *H. sapiens* macrophages. However, diversity in substrate preferences apparently has not increased after the separation of the Arthropoda and Chordata phyla, and the animal phosphoproteome was established early in animal evolution. This observation corresponds well with analyses of the animal phosphoproteome employing the primary sequences of kinases from divergent animals, as well as with very recent data showing that all major signalling pathways are present in the Porifera phylum, which separated from other animals very early in animal evolution [30], [31]. Lysates obtained from the fungal kingdom show much more diversity in array phosphorylation than animal lysates, with a *P. pastoris* lysate actually clustering with plants rather than with other members of the fungal kingdom. A possible explanation is that fungi consist of a diverse group of organisms closely related to plants [32], [33]. It must be noted however, that the other two fungi in the set are also not clustered together, again indicating a large diversity. The diversity in the phosphoproteomes can of course also be caused by the changes in evolutionary pressure on the different samples. It is possible that the evolutionary pressure on metabolic processes in organisms like fungi is of a different level when compared to plants or animals. When the average phosphorylation patterns of the plant, fungal, and animal kingdoms were compared (Figure 2C), the phosphorylation pattern of plants was found to more closely resemble the animal phosphorylation pattern than the fungal pattern.

### Extraction of a minimal phosphoproteome

The clustering analysis indicated that a significant subset of peptide substrates has remained evolutionarily stabile in terms of phosphorylation, irrespective of the eukaryotic source of the cell lysate. Hence, we decided to investigate the set of substrates whose phosphorylation is shared by all organisms tested in the present study. It appeared that phosphorylation of a set of 128 substrates was common to all organisms tested (If phosphorylation is random, one would expect only 0.6 substrates common in different tissues (binomial distribution 13 positive, 1152 total, cumulative chance 0,02; *p*<0.01) (supplementary information in Table S6). Table 3 lists the set of substrates that are phosphorylated by the divergent eukaryote cell lysates tested. Some of the substrates in the set are highly similar, *e.g.*, 12 slightly different peptides containing Ser15 of glycogen phosphorylase that were apparently deposited in PhosphoBase as separate substrates. When the list of pan-eukaryotic kinase targets is corrected for essentially identical peptide substrates, 71 different peptide substrates remained. These peptides are, in our set, the substrates for what may be termed a minimal eukaryotic phosphoproteome.

**Table 3:** Unique substrates phosphorylated in the majority of the profiles tested (supplementary info). Distribution in other species and the conservation of each substrate are also indicated.

Sequence	Ph-Site	Put. Kinase	SwissProt	Protein	Homologues	Conserved
GQEVYVKKT	Y-992	auto	Q02763	Angiopoietin-1 receptor	vertebrate, yeast	similar (except yeast)
LEKKYVRRD	Y-706	auto	P09581	macrophage colony stimulating factor 1 receptor	mammal	highly similar
KQPIYIVME	Y-424	auto	P00541	Tyrosine-protein kinase transforming protein Fps	mammal, fly	highly similar
YKNDYYRKR	Y-2131	auto	P08941	Ros proto-oncogene tyrosine kinase	vertebrate, yeast, worm	divergent
FKAFSPKGS	S-597	CDK	P12957	Caldesmon	aves	highly similar
EFPLSPPKK	S-37	CDK	P16949	stathmin	mammal, insect	similar
VIKRSPRKR	S-646	CDK	P08153	transcriptional factor SWI5	yeast, mammal	divergent
NWHMTPPRK	T-316	CDK	P13681	serine/threonine protein phosphatase PP1	bacterial, yeast	divergent
KISITSRKA	T-36	ERA	P06616	GTP-binding protein era	insect	-
DSTYYKASK	Y-577	FAK	P34152	Focal adhesion kinase	mammal, amphibian	highly similar
AKRISGKMA	S-277	G1/S kinase ?	P13863	Cell division control protein 2	mammal	highly similar
AVVRTPPKS	T-231	GSK3	P10636	Microtubule-associated protein tau	mammal	highly similar
VKRISGLIY	S-47	H4-PK-I	P02304	Histone H4	universal	highly similar
KGTGYIKTE	Y-701	JAK,Src	P42224	Signal Transducer and Activator of Transcription 1	mammal	highly similar
KNIVTPRTP	T-94	MAPK	P02687	Myelin basic protein	mammal, amphibian	highly similar
ELILSPRSK	S-24	MAPK,CDK	P16949	stathmin	mammal, insect	similar
AKKMSTYNV	S-315	MHCK	P19706	myosin heavy chain	mammal	-
KRAQISVRGL	S-15	PhK	P11217	glycogen phosphorylase	mammal	similar
TKKTSFVNF	S-218	PKA	P41035	eukaryotic translation initation factor 2 beta	mammal, plant, yeast, insect	highly similar
SRRQSVLVK	S-715	PKA	Q13002	glutamate receptor 6	mammal, amphibian	similar
RKASRKE	S-32	PKA	P02277	Histone H2B	mammal, shark	divergent
KRKRSRKES	S-32	PKA	P02278	Histone H2B	chordata	highly similar
KRFGSKAHM	S-374	PKA	P29476	nitric-oxide synthase	mammal	highly similar
EIKKSWSRW	S-467	PKA	P25107	parathyroid hormone/parathyroid hormone-related peptide receptor	mammal, yeast, funghi	divergent
KRRSSSYHV	S-687	PKA	P04775	Sodium channel protein type II alpha	mammal, squid	similar
KRKSSQALV	S-15	PKA	P03373	Transforming protein erbA	aves	divergent
RAKRSGSV	S-27	PKA	P12798	phosphorylase b kinase beta	mammal	similar
KKKKASVA	S-43	PKA	P12928	pyruvate kinase	-	-
KRRGSVPIL	S-247	PKA	P16452	erythrocyte membrane protein band 4.2	mammal, yeast	divergent
KLRRSSSVG	S-381	PKA,PKC	P02718	Acetylcholine receptor protein delta	fish	unique
KTRSSRAGL	S-19	PKA,PKC	P02261	Histone H2A	universal	highly similar
KRPSVRAKA	S-10	PKA,PKC	P02687	Myelin basic protein	mammal, amphibian	highly similar
GGRASDYKS	S-131	PKA,PKC	P02687	Myelin basic protein	mammal, amphibian	highly similar
KRKNSILNP	S-700	PKA,PKG	P13569	cystic fibrosis transmembrane conductance regulator	mammal	highly similar
TRIPSAKKY	S-104	PKC	Q62048	astrocytic phosphoprotein PEA-15	mammal	highly similar
KTTASTRKV	S-790	PKC	P13569	cystic fibrosis transmembrane conductance regulator	mammal	highly similar
RKAASVIAK	S-43	PKC	P06764	DNA polymerase beta	mammal, amphibian	highly similar
KKRLSVERI	S-29	PKC	P11388	DNA topoisomerase II alpha	mammal	highly similar
RGKSSSYSK	S-577	PKC	P02671	Fibrinogen alpha	human	-
STLASSFKR	S-889	PKC	Q05586	glutamate (NMDA) receptor subunit zeta 1	mammal, plant	similar
RVRKTKGKY	T-710	PKC	P19490	glutamate (NMDA) receptor subunit zeta 1	mammal, plant	similar
GGSVTKKRK	T-416	PKC	P11516	Lamin A/C	mammal, worm	divergent
KKKFSFKKP	S-92	PKC	P28667	MARCKS-related protein	mammal, aves	highly similar
AKDASKRGR	S-181	PKC	P10522	myelin	mammal, aves	highly similar
KRPSKRAKA	S-7	PKC	P02687	Myelin basic protein	mammal, amphibian	highly similar
KRAKAKTAKKR	T-9	PKC	P02612	Myosin regulatory light chain 2	mammal, aves, mussel	similar
SSKRAKAK	S-1	PKC	P02612	Myosin regulatory light chain 2	mammal, aves, mussel	similar
LSGFSFKKS	S-162	PKC	P30009	myristolated alanine-rich C-kinase substrate	mammal, aves	highly similar
LSGFSFKKN	S-169	PKC	P29966	myristolated alanine-rich C-kinase substrate	mammal, aves	highly similar
KKRFSFKKS	S-157	PKC	P12624	myristolated alanine-rich C-kinase substrate (MARCKS)	mammal	highly similar
GDKKSKKAK	S-23	PKC	P06685	Na+/K+ ATPase 1	mammal, bacteria	divergent
KIQASFRGH	S-36	PKC	P35722	neurogranin	vertebrate	divergent
KGQESFKKQ	S-227	PKC	P06748	Nucleophosmin	mammal	highly similar
KKLGSKKPQ	S-1506	PKC	P04775	Sodium channel protein type II alpha	mammal, bacteria	divergent
KSKISASRK	S-43	PKC	P08057	troponin I	mammal, aves, amphibian	highly similar
KAKVTGRWK	T-280	PKC	P13789	troponin T	mammal	highly similar
ALGISYGRK	S-46	PKC	P04326	TAT protein	viral	similar
RVRKSKGKY	S-717	PKC,PKG	P19491	glutamate receptor 2	mammal, insect	similar
FRKFTKSER	T-84	PKG	P00516	cGMP-dependent protein kinase (PKG)	mammal, yeast	similar
GAFSTVKGV	T-489	RK	P28327	rhodopsin kinase	mammal	highly similar
SRRPSYRKI	S-133	S6K	P16220	cAMP response element binding protein	mammal	highly similar
KASASPRRK	S-29	sperm-specific	P02256	Histone H1	sea urchin	highly similar
KRAASPRKS	S-10	sperm-specific	P02256	Histone H1	sea urchin	highly similar
KGGSYSQAA	Y-344	Src	P01889	HLA class I histocompatibility antigen B7	mammal	highly similar
TPAISPSKR	S-99	unknown	P33316	deoxyuridine 5′-triphosphate nucleotidohydrolase	human	-
KKDVTPVKA	T-53	unknown	P10156	Histone H1	bacteria	-
KSPAKTPVK	S-766	unknown	P19246	Neurofilament triplet H protein	mammal	-
KKASFKAKK	S-351	unknown	Q11179	Serine/threonine-protein kinase C	amphibian, mammal	divergent
SSLKSRKRA	S-39	unknown	P22613	spermatid nuclear transition protein 1	mammal	highly similar
KYRKSSLKS	S-35	unknown	P22613	spermatid nuclear transition protein 1	mammal	highly similar
GSLKSRKRA	S-39	unknown	P17306	spermatid nuclear transition protein 1	mammal	-

Remarkably, all substrates in table 3 contain one or more lysines (K), suggesting a bias in sequence composition or kinase. However, this seems unlikely as studies by Brinkworth *et al.* showed that, when using prediction models for substrates of kinases, the basic amino acids lysine (K) and arginine (R) are often required for optimal recognition of substrates [34]. Therefore the fact that lysine and arginine are present in the substrates in table 3 is not completely unexpected. Furthermore, it must be noted that the annotation of the substrates is based on the available data at present, and therefore incomplete. Profiling fungal lysates on a primarily mammalian set of substrates can cause the phosphorylation of irrelevant motifs. However the fact that these motifs are still phosphorylated clearly indicates the possible presence for kinase↔substrate interactions in other organisms even though no direct *in vivo* relevance is apparent. Table 4 shows the distribution of peptide substrates with regard to the molecular functions of their source proteins (according to Gene Ontology, based on human homologues in the Swiss-Prot database whenever possible). These data suggest that the phosphorylation events of this minimal phosphoproteome are associated with cell homeostasis; DNA replication, organisation, and stability; RNA translation; cytoskeletal organisation; motility; transmembrane ion transport; and signal transduction. Indeed, these are functions associated with every eukaryotic cell. When all of the peptides on the chip were subjected to a Blastp search (results are listed on http://www.koskov.nl), not all of the peptides included in the minimal kinome scored higher (*p*<0.01) for multiregnal homology hits than peptides not present in the pan-eukaryotic kinase substrate set. A possible explanation for this observation is that knowledge of non-mammalian regulation of phosphorylation is not as elaborate as that in mammals.

**Table 4:** Gene Ontology according to the Swiss-Prot database for the substrates of the minimal kinome, shown for humanized substrate set.

SwissProt	Protein	Biological Process	Molecular Function
P02718	Acetylcholine receptor protein subunit delta precursor	muscle contraction, signal transduction, transport	nicotinic acetylcholine-activated cation-selective channel activity
Q02763	Angiopoietin-1 receptor	cell-cell signaling, signal transduction, transmembrane receptor protein tyrosine kinase signaling pathway	protein kinase activity, receptor activity, transmembrane receptor protein tyrosine kinase activity
Q62048	Astrocytic phosphoprotein PEA-15	anti-apoptosis, negative regulation of glucose import, regulation of apoptosis, transport	protein binding
P12957	Caldesmon	Cel Motility	actin, tropomyosin, calmodulin binding
P16220	cAMP response element binding protein	signal transduction, DNA-dependent transcription	protein binding, transcription cofactor activity, transcription factor activity
P13863	Cell division control protein 2	apoptosis, cell proliferation, mitosis, protein amino acid phosphorylation, regulation of cell growth, regulation of mRNA processing, regulation of progression through cell cycle, regulation of transcription, DNA-dependent	ATP binding, protein binding, protein serine/threonine kinase activity
P13569	Cystic fibrosis transmembrane conductance regulator	respiratory gaseous exchange, transport	ATP binding, ATP-binding and phosphorylation-dependent chloride channel activity, channel-conductance-controlling ATPase activity, PDZ domain binding, protein binding
P33316	deoxyuridine 5′-triphosphate nucleotidohydrolase	DNA replication, nucleic acid metabolism	dUTP diphosphatase activity
P06764	DNA polymerase beta	DNA repair, DNA-dependent DNA replication	DNA polymerase activity, microtubule binding
P11388	DNA topoisomerase II alpha	DNA repair, DNA replication, signal transduction, regulation of apoptosis	DNA topoisomerase activity, drug binding, protein kinase C binding
P41035	Eukaryotic translation initiation factor 2 subunit beta	translational initiation	RNA binding, translation factor activity, nucleic acid binding
P02671	Fibrinogen alpha chain precursor	blood coagulation, tissue regeneration	extracellular region, fibrinogen complex
P34152	Focal Adhesion Kinase 1	cell motility, nucleus localization, extracellular matrix organization, microtubule cytoskeleton organization, negative regulation of organ size, neuron migration, signal transduction	protein binding, kinase activity
P35437	Glutamate [NMDA] receptor subunit zeta 1	calcium ion homeostasis, cation transport, glutamate signaling pathway, learning and/or memory, regulation of synaptic plasticity, response to ethanol, synaptic transmission	glutamate binding, glycine binding, glycine-gated ion channel activity, motor activity, N-methyl-D-aspartate selective glutamate receptor activity
Q05586	Glutamate (NMDA) receptor subunit zeta 1	signal transduction, transcription DNA-dependent	
P19491	Glutamate receptor 2	protein localization, positive regulation of synaptic transmission, receptor internalization, regulation of receptor recycling, regulation of synaptic transmission, glutamatergic, response to lithium ion, synaptic transmission	PDZ domain binding, protein binding, receptor activity
Q13002	Glutamate receptor 6	glutamate signaling pathway, synaptic transmission, transport	kainate selective glutamate receptor activity
P11217	Glycogen phosphorylase	glycogen metabolism	glycogen phosphorylase activity
P06616	GTP-binding protein era	growth control	GTP/GDP bindin
P02256	Histone H1	chromosome organization and biogenesis, nucleosome assembly	DNA binding
P10156	Histone H1	chromosome organization and biogenesis, nucleosome assembly	DNA binding
P02261	Histone H2A	nucleosome assembly	DNA binding
P02278	Histone H2B type 1	nucleosome assembly	DNA binding
P02277	Histone H2B	nucleosome assembly	DNA binding
P02305	Histone H4	establishment and/or maintenance of chromatin architecture, phosphoinositide-mediated signaling	DNA binding
P01889	HLA class I histocompatibility antigen	immune response	MHC class I receptor activity
P11516	Lamin A/C	nuclear membrane organization and biogenesis	protein binding
P09581	Macrophage colony-stimulating factor 1 receptor	antimicrobial humoral response, cell proliferation, development, signal transduction	macrophage colony stimulating factor receptor activity
P28667	MARCKS-related protein	cell motility, signal transduction	actin filament binding, calmodulin binding
P10636	Microtubule-associated protein tau	microtubule cytoskeleton organization and biogenesis, negative regulation of microtubule depolymerization, generation of neurons, positive regulation of axon extension, positive regulation of microtubule polymerization	enzyme binding, lipoprotein binding, microtubule binding, SH3 domain binding, structural constituent of cytoskeleton
P10522	Myelin	synaptic transmission	structural molecule activity
P02687	Myelin basic protein (MBP)	central nervous system development, immune response, nerve ensheathment, synaptic transmission	structural constituent of myelin sheath
P19706	Myosin heavy chain IB	actin filament-based movement	microfilament motor activity
P02612	Myosin regulatory light chain 2	actin filament-based movement, smooth muscle contraction	ATPase activity, calcium ion binding, microfilament motor activity
P12624	Myristoylated alanine-rich C-kinase substrate	cell motility	actin filament binding, calmodulin binding
P29966	Myristoylated alanine-rich C-kinase substrate	cell motility	actin filament binding, calmodulin binding
P30009	Myristoylated alanine-rich C-kinase substrate	cell motility	actin filament binding, calmodulin binding
P06685	Sodium/potassium-transporting ATPase alpha-1 chain precursor	ATP hydrolysis coupled proton transport, hydrogen ion homeostasis, potassium ion import, sodium ion transport, sperm motility	sodium:potassium-exchanging ATPase activity
P19246	Neurofilament triplet H protein	intermediate filament cytoskeleton organization and biogenesis	structural constituent of cytoskeleton
P35722	Neurogranin	nervous system development, signal transduction	calmodulin binding
P29476	nitric-oxide synthase	muscle contraction	nitric-oxide synthase activity
P06748	Nucleophosmin (NPM)	activation of NF-kappaB transcription factor, anti-apoptosis, cell aging, centrosome cycle, intracellular protein transport, negative regulation of cell proliferation, nucleocytoplasmic transport, response to stress, ribosome assembly, signal transduction	NF-kappaB binding, protein heterodimerization activity, protein homodimerization activity, RNA binding, Tat protein binding, transcription coactivator activity, unfolded protein binding
P25107	Parathyroid hormone/parathyroid hormone-related peptide receptor	G-protein signaling, coupled to cyclic nucleotide second messenger, skeletal development	parathyroid hormone receptor activity
P28327	Rhodopsin Kinase	regulation of G-protein coupled receptor protein signaling pathway, rhodopsin mediated signaling	protein kinase activity
P08941	Proto-oncogene tyrosine-protein kinase ROS	signal transduction	protein-tyrosine kinase activity, receptor activity
Q11179	Serine/threonine-protein kinase C	unknown	protein kinase activity
P42224	Signal transducer and activator of transcription 1-alpha/beta	caspase activation, I-kappaB kinase/NF-kappaB cascade, regulation of progression through cell cycle, response to pest, pathogen or parasite, signal transduction, transcription from RNA polymerase II promoter, tyrosine phosphorylation of STAT protein	hematopoietin/interferon-class (D200-domain) cytokine receptor signal transducer activity, protein binding, transcription factor activity
P04775	Sodium channel protein type 2	generation of action potential, sodium ion transport	Voltage-gated sodium channel activity
P17306	Spermatid nuclear transition protein 1	chromatin remodeling, chromatin silencing, fertilization, exchange of chromosomal proteins, nucleosome disassembly, sexual reproduction, single strand break repair, sperm motility, spermatid nuclear elongation	DNA binding
P22613	Spermatid nuclear transition protein 1	chromatin remodeling, chromatin silencing, fertilization, exchange of chromosomal proteins, nucleosome disassembly, sexual reproduction, single strand break repair, sperm motility, spermatid nuclear elongation	DNA binding
P16949	Stathmin	intracellular signaling cascade, microtubule depolymerization, mitotic spindle organization and biogenesis	signal transducer activity, tubulin binding
P03373	Transforming protein erbA	transcription from RNA polymerase II promoter	protein binding, thyroid hormone receptor activity, transcription factor activity
P08153	Transcriptional Factor SWI5	G1-specific transcription in mitotic cell cycle	transcriptional activator activity
P08057	Troponin I	negative regulation of angiogenesis, regulation of heart contraction, regulation of muscle contraction	actin binding, calcium channel inhibitor activity, protein binding
P13789	Troponin T	regulation of muscle contraction	protein binding, tropomyosin binding
P00541	Tyrosine-protein kinase transforming protein Fps	cell proliferation, development, protein amino acid phosphorylation	protein-tyrosine kinase activity

For most substrates in this minimal phosphoproteome set, a kinase capable of phosphorylating the peptide has been described (Table 3). Although most of the kinases in this list are common to all eukaryotes (*e.g.*, phosphorylase kinase and S6 kinase), some are unique to animals. This is especially true for the tyrosine kinases Src, Ros, and c-Fms, which do not have orthologues in plants or fungi. Hence, phosphorylation of tyrosine in the substrates by plant or fungal cell lysates proceeds through other kinases that have similar substrate specificities as the members of the tyrosine kinase family in animals. Possible candidates for such phosphorylation are the kinases belonging to the dual specificity DYRK, STE7, and Wee family of kinases, which are thought to be capable of tyrosine phosphorylation [35]–[38]. However, unique groups of kinases in these species could also be candidates. Interestingly, a recent analysis of the *D. discoideum* kinome identified a number of kinases that, based on their primary sequences, may act as tyrosine kinases [18]. In *A. thaliana*, APK1 is capable of tyrosine phosphorylation [13]. It would be interesting to investigate whether any of these kinases are responsible for this minimal phosphoproteome tyrosine phosphorylation events observed in the present study. Interestingly, inhibitors of animal tyrosine kinases also function in plants, suggesting substantial structural homology between the kinases responsible for tyrosine phosphorylation in both kingdoms [39], [40]. Further insights into kinase evolution and specificity in different species are needed.

### Peptides in this minimal phosphoproteome are not general kinase substrates

An important question concerns the necessity of this minimal eukaryotic phosphoproteome for cell function. The finding that a set of peptide substrates is phosphorylated by cell lysates from highly divergent eukaryotes may indicate that such kinase activity is essential for eukaryotic life and that strong evolutionary pressure exists to prevent its loss. An alternative explanation would be that these substrates act as so-called über-substrates that are relatively non-specifically phosphorylated by multiple kinases. To investigate this question, we incubated chips with relatively high concentrations of purified kinases, *e.g.*, human Tpl2 (MAP3K8). We observed that the substrates phosphorylated by these purified kinases did not overlap with the set of substrates comprising this minimal eukaryotic phosphoproteome (*R*2 = 0.11). Thus, phosphorylation of the substrates in the minimal phosphoproteome likely reflects the specific activities of multiple kinases in the eukaryotic cell lysates. However, this can only be validated when the phosphorylation profile all kinases are analysed separately. Apparently, strong evolutionary pressure on a minimal phosphoproteome exists, counteracting changes in substrate specificity for the kinases responsible for these phosphorylation events. By inference, this set of substrate motifs was probably present in an ancestral eukaryotic progenitor cell. This notion is in agreement with a recent study by Scheeff and Bourne provides convincing evidence for the evolution of the various kinase families from a common ancestor [41]. It is tempting to speculate that this ancestral protein kinase, or other kinases that appeared relatively early in the history of eukaryotic life, delivered the foundation of essential kinase substrate motifs (the minimal eukaryotic phosphoproteome) that remained stabile ever since.

Concluding, in this paper we described the presence of a set of kinase substrates that is recognised and phosphorylated by a diverse panel of eukaryotic cell lysates. This is remarkable since this set is biased towards mammalian motifs, but can still be a target of non mammalian lysates. The fact that this occurs indicates that some level of conservation exists in the eukaryotic linage. Analysis of the preferred substrates revealed that lysine and arginine have an important role in primary sequence of kinase substrates. The possibility that the minimal kinome is produced by a few kinases seems unlikely since single kinase experiments reproduce a very limited part of this panel. However a limited set of kinases can very well be able to reproduce this set. This seems not unlikely since the major function of this set is to maintain cell homeostasis, other more specialised functions require specialised kinases.

## Materials and Methods

### Organisms

Whole extracts of *C. albicans*, *P. pastoris*, *F. Solani*, *D. melanogaster*, *T. aestivium* and *A. thaliana* were used and cell types of *M. musculus* and *H. sapiens* were used as mentioned in the text.

### Peptide Array Analysis

For kinome array samples, 10^6^ ceq or 500 µg were lysed or homogenised in 100 µl of cell lysis buffer (20 mM Tris-HCl, pH 7.5, 150 mM NaCl, 1 mM Na2EDTA, 1 mM EGTA, 1% Triton X-100, 2.5 mM sodium pyrophosphate, 1 mM MgCl2, 1 mM β-glycerophosphate, 1 mM Na_3_VO_4_, 1 mM NaF, 1 µg/ml leupeptin, 1 µg/ml aprotinin, 1 mM PMSF). The cell lysates were subsequently cleared on a 0.22-µm filter. Peptide array incubation mix was produced by adding 10 µl of filter-cleared activation mix (50% glycerol, 50 µM [γ-33P] ATP, 0.05% v/v Brij-35, 0.25 mg/ml bovine serum albumin, [γ-33P] ATP (1000 kBq)). Next, the peptide array mix was added onto the chip, and the chip was kept at 37°C in a humidified stove for 90 min. Subsequently the peptide array was washed twice with Tris-buffered saline with Tween, twice in 2 M NaCl, and twice in demineralized H_2_O and then air-dried. The experiments were performed three times in duplicate.

### Analysis of Peptide Array

The chips were exposed to a phosphorimager plate for 72 hours, and the density of the spots was measured and analyzed with array software.

### Analysis

For the analysis clustering using the spearman correlation coefficient was calculated for each combination of sets and clustering was performed using Johnston hierarchical clustering schemes. Inclusion parameters for each of the kinome profiles are described in supplemental data, Table S4.

## Supporting Information

Table S1 Substrates spotted on the PepChip.
(0.17 MB XLS)

Table S2 List of monophospho-acceptor motifs, with the distribution of histidine and aspartate residues indicated.
(0.11 MB XLS)

Table S3 Presence (1) or absence (0) of spots phosphorylated by the different lysates tested. This table is used to determine the clustering of the different lysates.
(0.32 MB XLS)

Table S4 Presence (1) or absence (0) of spots phosphorylated averaged for the different sample background.
(0.23 MB XLS)

Table S5 Presence (1) or absence (0) of spots phosphorylated averaged for the different organisms.
(0.25 MB XLS)

Table S6 Calculation of the probability that 116 trials (substrates) are positive (in at least 90% of the samples, corrected for origin bias) in a total number of 1152 trials ( = whole PepChip) using a binominal distribution calculation (http://www.stat.sc.edu/∼west/applets/binomialdemo.html). The p-value for success in the binomial distribution is calculated by using the cumulative relative amount of positive spots for every organism. The result of this test shows the chance that a spot is phosphorylated in every set.
(0.32 MB XLS)

Histogram S1 Histogram of frequency distribution of Table S3.
(0.02 MB XLS)

Histogram S2 Histogram of frequency distribution of Table S4.
(0.02 MB XLS)

Histogram S3 Histogram of frequency distribution of Table S5.
(0.03 MB XLS)
